# Dengue Outbreak in a Hilly State of Arunachal Pradesh in Northeast India

**DOI:** 10.1155/2014/584093

**Published:** 2014-01-21

**Authors:** Siraj A. Khan, Prafulla Dutta, Rashmee Topno, Monika Soni, Jagadish Mahanta

**Affiliations:** Entomology and Filariasis Division, Regional Medical Research Centre, (ICMR), NE Region, P.O. Box 105, Dibrugarh, Assam, India

## Abstract

Dengue has been reported from plains as well as hilly regions of India including some parts of Northeast India. In July-August 2012, outbreak of fever with unknown origin (FUO) indicative of Dengue was reported in Pasighat, East Siang district of Arunachal Pradesh (AP) state. Serum samples (*n* = 164) collected from patients from Health Training and Research Centre General Hospital, Pasighat, were tested for NS1 antigen and IgM antibodies. NS1-positive samples were analyzed by RT-PCR assay and entomological surveys were carried out. The majority of suspected cases reported NS1 antigen positivity. Females and young adults were mostly affected. The majority of the amplified NS1-positive samples showed Dengue serotype 3 infection. *Aedes* (Stegomyia) *albopictus*, known as semiurban breeding mosquitoes, was the only potential vector species identified from the affected areas of Pasighat which single handedly contributed to the outbreak. Thus, the present work identifies Dengue as an emerging arboviral infection in hilly state of AP along with a looming risk of its spread to neighbouring areas.

## 1. Introduction

Dengue is a mosquito-borne viral disease of global public health concern. More than 100 countries in Africa, America, Eastern Mediterranean, Southeast Asia, and Western Asia are affected. The disease poses a threat to more than 1.8 billion people in the tropics and subtropical region infecting about 100 million people every year [1]. According to World Health Organization (WHO), Dengue is the fastest spreading tropical disease and represents a pandemic threat [2]. Dengue viral infection may be asymptomatic or may give rise to undifferentiated fever with or without other associated clinical manifestations, namely, Dengue fever (DF), Dengue hemorrhagic fever (DHF), or Dengue shock syndrome (DSS) [3]. Dengue is caused by Dengue virus (DENV) that comprises of four serotypes (DENV 1–4). DENV is a single-stranded, positive sense RNA, enveloped virus belonging to genus *Flavivirus* under family *Flaviviridae*. Dengue transmission in humans is caused by *Aedes* (Stegomyia) *aegypti *(Linnaeus) and *Aedes* (Stegomyia) *albopictus* (Skuse) mosquito species [4].

In India, Dengue was first reported in 1945 [5]. Thereafter, at a gap of 18 years, in 1963-1964, an initial epidemic of DF was reported on the Eastern Coast of India. The disease spread northwards and reached Delhi and Kanpur in 1967 and 1968 respectively. Simultaneously, it also involved the southern part of the country and gradually the entire country was involved with widespread epidemics followed by endemic/hyperendemic prevalence. Rapid growths of population and urbanisation compounded by change in climate have contributed significantly towards the increase in cases of DF/DHF in India [4, 6]. Dengue is no more an urban area infection but it is extending to rural areas also [7]. The disease has recently spread to hilly regions like Nilgiris and Cardamom hills as well. 20 serologically positive patients were reported from Coimbatore, located at an altitude of 411 metres (m) above mean sea level (MSL) on the eastern slopes of Nilgiri Hills in 1998 [8].

In the northeast region (NER) of India, serological survey conducted during 1963 revealed Dengue activity in the Lohit district of Arunachal Pradesh (AP) and Darrang district of Assam [9, 10]. Subsequently, another report of Dengue (DENV-2) in Assam and Nagaland appeared during the nineties [10–12]. During 2009–2011, a study carried out by Dutta et al. (2012) reported 143 laboratory confirmed cases belonging to Assam (82), Meghalaya (35), Nagaland (15), Manipur (8), and AP (3) [13].

In the present study, we report Dengue outbreak in Pasighat, a hill station located at an altitude of 155 m MSL (latitude: 28.07° N, longitude: 95.33°E) situated in the East Siang district of AP. The outbreak was confirmed by serological, clinical, and molecular evidence.

## 2. Materials and Methods

### 2.1. The Outbreak Study

During the months of July-August 2012, suspected cases of fever of unknown origin (FUO) were reported from Pasighat, AP, India. The case definitions of suspected Dengue infection were based on guidelines of Centers for Disease Control and Prevention (CDC). The clinical descriptions include fever and two or more of the following: headache, retroorbital pain, myalgia, arthralgia, rash, hemorrhagic manifestations, or leucopenia. A total of 164 probable patients from hospitalised/Out Patient Department (OPD) at Health Training and Research Centre General Hospital, Pasighat, conforming to the above mentioned case definition were recorded. Serum samples collected from the patients were transported under cold conditions to the Regional Medical Research Centre (RMRC), ICMR, NE Region, Dibrugarh, India, for laboratory investigations. The nonresearch activity was a part of public health response to outbreak investigation and thus did not require a review of Institutional Ethics Committee of RMRC, ICMR, Dibrugarh.

### 2.2. Serological Diagnosis

All serum samples were tested for presence of Dengue viral nonstructural 1 (NS1) antigen by using Platelia Dengue NS1 kits (Bio-Rad, USA). The samples were also tested for Dengue specific immunoglobulin M (IgM) antibody by using IgM antibody capture enzyme-linked immunosorbent assay (MAC-ELISA) kits procured from the National Institute of Virology (NIV), Pune, India. NS1-positive samples were also tested for IgG using DENGUE IgG capture ELISA (Panbio, Australia). All tests were carried out according to manufacturer's instructions.

### 2.3. Molecular Typing

Dengue NS1-positive serum samples were subjected to detect Dengue specific RNA by two-step reverse transcriptase polymerase chain reaction (RT-PCR) assay based on Harris et al., 1998 [14]. Viral RNA was extracted using QIAamp viral RNA mini kit (Qiagen, Germany). Complementary DNA (cDNA) was synthesised using cDNA synthesis kit (Fermentas, USA). PCR was carried out using primer pairs amplifying capsid region according to Lanciotti et al., 1992 [15], using cDNA as template. Amplified PCR products were visualized in 2% agarose gel electrophoresis.

### 2.4. Follow-Up Survey of Dengue Affected Areas and Vector Monitoring

Following laboratory confirmation of Dengue outbreak, an entomological survey was undertaken to identify the vector(s) involved in order to devise vector control and disease containment strategies. As part of mosquito surveillance program, solid waste containers, namely, bamboo stumps, metal and plastic wares, cans, flower pots, discarded automobile tyres, and so forth, whichever could hold rain water or were used for water storage, were searched for immature stages (larvae and pupae) of mosquitoes. Immature mosquitoes were link reared and, on emergence to adults, were identified using standard mosquito identification keys [16]. Adult mosquitoes were screened for virus detection according to the above mentioned protocol. Fifty-six adult mosquitoes of *Aedes* species were collected from indoors and scrape yards.

## 3. Results

### 3.1. Serological Findings

Out of 164 samples, 107 (65.2%) were found to be Dengue positive. Ninety-eight (91.5%) NS1 positives, 5 (4.6%) IgM positives, and 4 (3.7%) both NS1 and IgM positives were detected. To test the recency of outbreak, 87 NS1-positive samples were tested for IgG antibodies and 5 positives were detected (6%). One sample was positive for all the tests: NS1, IgM, and IgG. Four samples were both NS1 and IgG positive but IgM negative. Clinical profiles for all positive cases are summarized in Table 1. The common clinical presentations were fever (98.1%), headache (93.4%), myalgia (81.3%), arthralgia (52.3%), vertigo (41.1%), and retroorbital pain (28.0%). Female patients comprised 67.2% of the positive cases. The positive patients were from all age groups (range 9–70 years) with a median age of 28 years. 46.7% of the cases belonged to the young adult age group (18–30 years).

### 3.2. Molecular Typing of Dengue Serotypes

Eighty-nine of the 98 NS1-positive samples were processed for Dengue-specific two-step RT-PCR assay. PCR amplification was detected in 35 samples (39.3%) of which 27 (77%) were Dengue serotype 3. Seven (20%) samples were coinfected by serotypes 1 and 3, whereas 1 (3%) sample had coinfection of serotype, 2 and 3. Thus, serotype 3 was the predominant serotype circulating during the outbreak.

### 3.3. Vector Surveillance

Following confirmation of Dengue outbreak, an entomological survey was carried out in the Pasighat area. Only *A. albopictus* mosquitoes were caught during the survey both in immature as well as adult collections. All the mosquitoes screened for Dengue virus were PCR negative. The most common habitats for *Aedes* breeding were discarded automobile tyres mostly stored in the open in tyre repairing shops and solid waste scrape dump yards in the vicinity of the market area; bamboo stumps in the residential localities as well as in the extensive bamboo plantations adjoining the human habitations and discarded plastic containers around houses having container index values of 57.1%, 42.8%, and 28.5%, respectively. Breteau Index (BI) for* A. albopictus* was estimated as 75.0.

## 4. Discussion

**Figure 1:**
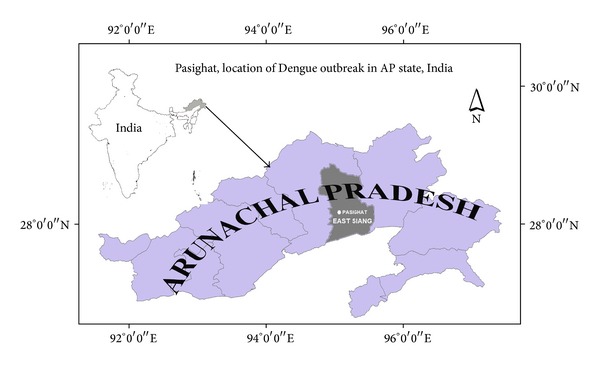
Pasighat, location of Dengue outbreak in AP state, India.

The present paper reports Dengue outbreak from Pasighat hill station (Figure 1), East Siang district of AP. The East Siang district had never reported any Dengue infection earlier.

It was observed that the majority of Dengue cases were detected by the presence of viral NS1 antigen compared to IgM antibodies in patient's sera. Therefore, it is known that early detection of Dengue cases by NS1 assay helps in diagnostic detection and confirmation of cases [17]. Viral antigen detection is particularly useful during the first five days of illness with NS1 assays that are significantly more sensitive for primary than secondary Dengue infection [18–20]. Six percent of NS1-positive samples were also IgG positive. These patients provided serological evidence of previous exposure. It might also be possible that IgG positivity in these patients could be due to early development of IgG response at the end of first week of acute Dengue infection rather than previous exposure to another Dengue serotype/infection. One patient positive for all: NS1, IgM, and IgG was probably in the late stage of either a primary or a secondary infection and might have been infectious for mosquitoes [21]. Clinical presentations of Dengue-positive cases showed that fever was the most common presenting symptom as it was also observed in other studies [22]. Other common symptoms found were headache [23], musculo-skeletal symptoms (arthralgia and myalgia) [24], retroorbital pain, and itching [25]. Thus, it can be stated that supporting clinical symptoms along with early detection of viral NS1 antigen can help to speedup diagnosis of Dengue cases.

DF is typically known as a childhood disease and is an important cause of paediatric hospitalisation in Southeast Asia [26]. However, in India, all age groups have been affected by DF [27]. In an outbreak in Delhi during 2003, dengue-positives in the adult group outnumbered those of children although the difference in the number of positive cases was not significant compared to pediatric age group [28]. In another outbreak in Malaysia during 2006-2007, it was observed that the majority of the cases were adults in the 21 to 25 years and >35 years old age groups, with mean percentages of 20.5% and 23%, respectively. This trend is similar in most dengue endemic countries in Southeast Asia. With this changing demography, it is possible that there are features of severe dengue leading to death that could be different from those seen in children [29]. In the present study, it was observed that the maximum cases were in the young adult age group; similar observations were also found in other studies [30]. Female cases were more than males; this observation tallied with another study carried out in Kolkata, India, in 2010 [31]. The vector mosquitoes (*Aedes sp.*) are mainly domestic and peridomestic in nature and females/house wives have a greater chance of exposure to mosquito bites.

For molecular typing of NS1-positive samples, RT-PCR assay was carried out. It was observed that only 39.3% of the cases showed molecular amplification among NS1-positive cases. It can be stated that in early detection, NS1 antigen detection appeared to be better than RT-PCR. In a study, DENV NS1 antigen detection in travelers upon arrival at airports in Taiwan was important in detection of 19 RT PCR-negative travelers who would have been labeled DENV negative [32]. Furthermore, in Malaysia, 42 of 55 patients with a diagnosis of acute Dengue were positive for NS1 but negative by both RT-PCR and virus isolation [33]. In India, during past Dengue epidemics, all four serotypes were found to circulate, but type 2 was the most predominant type. However, in recent trends, epidemiology of Dengue infection is changing in India with predominance of serotype 3 [34]. In our study also, serotype 3 was the major serotype circulating. Among the 35 PCR-positive samples, monotypic infections of type 3 were observed in 27 (77%) samples.

In entomological survey, among the potential Dengue vectors, only* A. albopictus* was found. The species has been implicated as an efficient potential vector of epidemic Dengue [35] although it is believed to be a less efficient vector of arboviruses than* A. aegypti*, the major reported vector of Dengue. However,* A. albopictus* adapts better than* A. aegypti* in temperate climate and outbreaks may be caused by this species of mosquitoes in temperate regions and also in areas where* A. aegypti* is not present [36]. But outbreaks caused by* A. albopictus* are usually smaller and mild in nature. In spite of being considered as a less efficient vector, its notoriety with regard to spread of dengue infection is increasing due to a rapid change in its overall distribution. In recent decade,* A. albopictus* has been the vector in outbreaks in different areas of the world, namely, China [37], Hawaii [38], and Mauritius [39]. The region of Pasighat experiences a temperate climate; warm summers and winters are moderately cold [40]. Abundance of* A. albopictus* from the area supported the role of the species in the Dengue outbreak. A Breteau index lower than 5 denotes a low risk, whereas an index value greater than 50 indicates a high risk of Dengue transmission [41]. We recorded a BI of 75 which made the area vulnerable to a high risk of Dengue transmission. However, none of the mosquitoes could be incriminated for the presence of Dengue viral RNA.* A. albopictus* is a mosquito of semiurban area [10]. This mosquito was observed in Pasighat, a semiurban town. Dengue is a disease of urban areas where solid wastes, air conditioners, air coolers, flower pots, and so forth are the major contributors in the growth of* A. aegypti*, the principal urban vector of Dengue. On the contrary, Pasighat being a semiurban town surrounded by lash green vegetation including bamboo plantations was devoid of* A. aegypti*. In this township located at an elevation of 155 MSL, bamboo plantations were found to be the major contributors supporting the breeding of* A. albopictus* mosquitoes. This species of mosquitoes single-handedly caused the Dengue outbreak. Mature bamboos are cut just at around internode having ample space for collection of rain water. The people were educated to cut the bamboo at the node or fill the already cut bamboo stamp with soil so as just to avoid water collection. The decline in breeding of* A. albopictus* was noticeable in four-week time.

Moreover, insecticide spraying, public health education (including community source reduction), and probably onset of dry winter season may have contributed to the culmination of the outbreak. Though Dengue is widely endemic in many parts of the world, there were no reports of Dengue cases from Pasighat region of the state. However, after the present outbreak, the disease has to be recognized as an emerging public health problem in the state. *A. albopictus* seemed to be the lone vector species responsible for the virus transmission. Public awareness activities combined with efforts to eliminate the identified risk factors for vector breeding could be instrumental in prevention of further outbreaks in the future.

## 5. Conclusions

The recent outbreak has established Dengue as an arboviral disease in the AP state, NE India, of public concern. Females and young adult groups were more affected. Molecular typing elucidated type 3 as the major circulating serotype. Entomological surveys conducted in the area during the outbreak have shown the presence of only one potential vector species, namely,* A. albopictus*, the mosquito of semiurban areas. Identification of etiological agent, timely intervention, and public awareness led to a prompt reduction in the intensity of outbreak. Information, education, and communication (IEC) activities combined with efforts towards vector breeding source reduction would go a long way in prevention of spread of the disease.

## Figures and Tables

**Table 1:** Clinical characteristics of Dengue-positive cases.

Clinical features	Percentage frequency
Fever	105 (98.1)
Headache	100 (93.4)
Myalgia	87 (81.3)
Arthralgia	56 (52.3)
Vomiting	46 (42.9)
Vertigo	44 (41.1)
Retroorbital pain	30 (28.0)
Itching eruption	28 (26.1)
Loose motion	20 (18.6)
Cough	20 (18.6)
Pain in abdomen	20 (18.6)
Rashes	18 (16.8)
Hypotension	18 (16.8)
Sore throat	17 (15.8)
Irritability	7 (6.5)
Nausea	4 (3.7)
Dysuria	3 (2.8)
